# Telephone-first access to general practice for older people: a qualitative study

**DOI:** 10.3399/BJGPO.2025.0133

**Published:** 2026-06-17

**Authors:** Joanne Parsons, Carol Bryce, Joanna Fleming, Jennifer Newbould, Jeremy Dale, Helen Atherton

**Affiliations:** 1 Centre for Evidence and Implementation Science, University of Birmingham, Birmingham, UK; 2 Warwick Applied Health, University of Warwick, Coventry, UK; 3 RAND Europe, Cambridge, UK; 4 Primary Care Research Centre, University of Southampton, Southampton, UK

**Keywords:** Consultation, Qualitative research, Triage, General practitioners, Primary healthcare

## Abstract

**Background:**

Recent years have seen increasing pressure on primary care workforce and appointments, with telephone-first introduced in the UK to manage demand and workload. Patients discuss healthcare needs via telephone with a GP before being invited to make an appointment.

Older people are at increased risk of inequality in accessing primary care appointments, with more long-term conditions and increased communication difficulties using telephone. These inequalities were potentially exacerbated during the COVID-19 pandemic.

**Aim:**

This study aimed to explore experiences of older people, carers, and general practice teams in using telephone-first to access appointments.

**Design & setting:**

Qualitative study in primary care.

**Method:**

We conducted 48 interviews with older people or their carers and six focus groups with staff from general practices using telephone-first.

**Results:**

Practices and older patients had varied approaches to telephone-first. As well as adapting to the concept of triage call-back, challenges for older people and their practices included changing their understanding of what constituted consultations. Trust between patients and their general practice influenced views and experiences, with acceptance of telephone-first being linked to their overall trust in the general practice. We observed differing views on how telephone-first worked between patients and general practices reflecting poor communication between the two groups.

**Conclusion:**

Systems implemented into practices need to be adequately explained, regarding processes, staff roles, and expectations of patients, to allow for thorough understanding and a demystification of the unknown. Future research should examine how telephone-first approaches affect older patients' health outcomes.

## How this fits in

Recent years have seen increasing pressure on primary care workforces and appointments, with telephone-first introduced in the UK as a way to manage demand and workload. There is limited evidence on its use and acceptance; this includes the impact on those with greater health needs, such as older people. This research described experiences of older people, carers, and general practice staff in using telephone-first, and described how clear explanations can help to both aid understanding and demystify systems and processes.

## Introduction

General practice is facing escalating pressure in the form of increased demand for appointments and fewer GPs.^1,2^ The telephone-first access system was introduced as a way to manage demand, allow easier contact with a GP, and reduce attendance in general practice and at hospital.^3^

Telephone-first requires patients wanting an appointment to first discuss their problem with a healthcare professional over the telephone. Either problems are resolved over the telephone or patients are booked in for a face-to-face appointment. General practices in the UK take an individual approach to choosing and applying any given access system. Telephone-first as an approach appeared in some general practices as long ago as 2011.^4^ Prior to the COVID-19 pandemic, there was a steady increase in the number of practices using telephone-first, however use sharply escalated from March 2020 when general practices needed to provide as much remote care as possible.^5^

Despite the increased use of telephone-first, there is limited evidence on its use and acceptance, this includes the impact on those with greater health needs. Increasing health needs are associated with ageing^6–8^ and older people are more likely than other age groups to need to access a GP.^9^ Between 2005 and 2019, rates of patients with 3 or more long-term conditions increased by over 6% and these are primarily older patients.^10^ Older people may encounter challenges when using telephone-first, such as needing a carer to receive a call-back or having sensory disabilities that make using a telephone difficult. This may present barriers to timely access to care,^11^ potentially leading to delays in receiving treatment, avoidable morbidity, and adverse impacts on quality of life.^12^

Research examining remote consultations (including online and varied telephone triage approaches) has identified that they compromise traditional human elements of general practice such as relationship-based care.^13^ Personalised relationships with the GP and continuity of care are important for older people.^14^ They view the relationship with a general practice as a long-term social contract, in which they do not waste the doctor’s time in return for goodwill in the form of flexibility in access and appointment systems.^15^ This may be challenged by the introduction of access systems such as telephone-first.^15^

This study aimed to explore the experience and perceptions of older people, their carers, and general practice teams in using a telephone-first approach for access to appointments, to better understand its impact.

## Method

This research was reported using the Standards for Reporting Qualitative Research framework.^16^ This qualitative study consisted of patient or carer interviews and focus groups with practice staff.

The study was conducted in 2020–2021 and designed and funded prior to the COVID-19 pandemic.

### Researcher characteristics and reflexivity

The research team consisted of four researchers working in health sciences. All were experienced qualitative researchers with experience in primary care topics and researching potentially sensitive topics.

Reflexivity was ensured by fortnightly discussion among the researchers during data collection and analysis. The wider research team had varied backgrounds and expertise, including healthcare professionals, and were involved in discussions about the meaning and interpretation of findings.

### Patient and public involvement (PPI)

PPI input occurred throughout the study; a lay co-applicant was in the research team. Input was received on patient-facing documents, interview schedules, and in analysis of data where they joined analysis meetings.

### Sample and recruitment

We sought to recruit eight general practices in the West Midlands region, varied by list size, location (urban or rural), and deprivation score. Recruited practices identified and invited eligible patients; we aimed to interview 5–6 patients per practice.

Patient participants were aged 65 or older in line with the Office for National Statistics categories for the UK census^17^ and had received a telephone-first telephone call-back appointment in the last month. We sought a maximally variable sample of participants in relation to: age group (65–69, 70–74, 75–79, and 80+), ethnic group, gender, and number of health conditions.

We sought to conduct focus groups in up to six of the eight general practices, aiming to recruit 4–6 staff members per practice (across roles, for example GPs, nurses, and administration staff) to each focus group. We aimed for variability in the roles of participating staff. We reimbursed practices for the time taken in participating in the focus groups, and six was the maximum number of focus groups that we could adequately fund to ensure full participation within a practice. We selected the six practices for maximum variability of characteristics (practice size, deprivation score, location, and proportion of patients in minority ethnic groups). Practices identified staff who were involved in or responsible for the telephone-first system and invited them to take part.

### Data collection

The topic guides for interviews and focus groups were developed by the research team, drawing on those from previous studies and in the literature (Supplemental Materials 1 and 2).^11,18,19^

The interview topic guide for patients explored experiences of telephone-first: any difficulties experienced, instances where it worked well, and instances where it did not work. Interviews were conducted via telephone (due to COVID-19 restrictions) and audio-recorded, transcribed, and anonymised.

The topic guide for staff focus groups explored how telephone-first had been implemented in the practice, how the system works for patients, any specific policies implemented for older people, and any perceived challenges or benefits for older patients using telephone-first. Focus groups were conducted over Microsoft Teams and were recorded, transcribed, and anonymised.

### Data analysis

Analysis used a codebook approach to thematic analysis.^20^ This allowed a flexible approach to analysis using a structured codebook, while also recognising the interpretative nature of data coding. Focus group data were analysed alongside patient data. Transcripts were read by several team members and an initial coding framework was devised. Initial themes from the interviews were discussed and the ‘one sheet of paper’ approach was used to develop one-page summaries that went on to form themes.^21^ Our PPI team member was involved in the analysis.

## Results

Eight general practices participated (see Table 1). Two were using telephone-first prior to March 2020; the remaining practices had implemented telephone-first in March 2020 as a result of the onset of the pandemic, with one of them having already been well advanced in their plans to introduce it in early 2020.

**Table 1.** Characteristics of included practices

	Date of adoption of telephone-first approach	Practice size Small (<6000), Medium (6000–12 000), Large (>12 000)	Location type	Deprivation index (IMD 2019)^a^	Ethnicity profile of practice location^31^
**Practice 1**	Pre-Dec 2019	Large	Urban	6	1.8% non-White ethnic groups, 98.2% White ethnic group
**Practice 2**	March 2020	Medium	Urban	5	1.3% non-White ethnic groups, 98.7% White
**Practice 3**	Pre-Dec 2019	Large	Urban	10	2.2% mixed, 5.0% Asian, 1.5% other non-White ethnic groups, 91.3% White
**Practice 4**	March 2020	Small	Urban	6	3.2% mixed, 16.8% Asian, 7.8% Black, 3% non-White ethnic groups, 69.2% White
**Practice 5**	March 2020	Small	Rural	6	2.2% non-White ethnic groups, 97.8% White
**Practice 6**	March 2020	Large	Urban	5	3.5% mixed, 27.4% Asian, 9.4% Black, 5.1% other non-White ethnic group, 54.6% White
**Practice 7**	Pre-pandemic planning, March 2020 implementation	Large	Rural	9	1.7% mixed, 1.6% Asian, 1.0% other non-White ethnic groups, 95.7% White
**Practice 8**	March 2020	Small	Urban	2	3.2% mixed, 21.7% Asian, 8.7% Black, 2.5% other non-White ethnic groups, 63.9% White

IMD = Index of Multiple Deprivation.

a 1 is highest deprivation level and 10 is lowest deprivation level.

Across the eight practices, 48 patients participated (see Table 2).

**Table 2.** Self-reported patient characteristics

Participant characteristics	Frequency
**Gender**	
Male Female	21 27
**Ethnic group**	
Asian Mixed White Other	6 4 37 1
**Age, years**	
65–69 70–74 75–79 ≥80	17 12 5 14
**Number of health conditions**	
3 or fewer conditions (multimorbidity)^32^ 4 or more conditions (complex multimorbidity)^33^	39 9
**Presence of disability**	
Yes No	13 35
**Number of medications prescribed**	
0 1–4 5–10 More than 10	4 18 20 6
**Carer**	
Yes Makes appointments for someone else No	3 8 37

Focus groups were conducted with six practices and these included staff with different roles, with a total of 29 focus group attendees (see Table 3).

**Table 3.** Characteristics of focus group attendees

	Attendees, *n*	Attendee roles
**Practice 1**	5	Receptionist, practice nurse, advanced nurse practitioner (visiting team), GP (*n* = 2)
**Practice 2**	7	Lead receptionist, receptionist, administrator, practice nurse, advanced nurse practitioner, GP, healthcare assistant
**Practice 3**	4	Receptionist, assistant practice manager, GP partner, physician associate
**Practice 5**	4	Reception manager, lead nurse, GP, GP locum
**Practice 6**	6	Practice nurse, nurse practitioner, GP (*n* = 3), practice manager
**Practice 7**	3	Reception manager, nurse prescriber, GP partner

### Setting the scene

Each practice was operationalising telephone-first differently: some practices offered short time slots for call-backs; others offered wider morning or afternoon slots in which the patient should expect a call-back. Some practices retained some ‘book ahead’ appointments, while others only had same-day appointments. As a result, there was variability in how patients were expected to engage with telephone-first.

### Themes

We identified five themes. Themes are explained below and illustrated with quotes from patients or carers and practice staff. A table of themes and sub-themes is also provided (Table 4).

**Table 4.** Themes and sub-themes

Theme	Sub-theme
Telephone-first comes into being	
Conceptualising the consultation	
Identities	Needs defined by age Identity as a carer
Trust as a driver for views and experiences	Trust in the system Trust in practice staff Trust in patients
Impact on roles	Roles of practice staff The patient role

#### Telephone-first comes into being

Practices shared how they came to use telephone-first, and for several this was due to the COVID-19 pandemic.

One practice had been using telephone-first for 5 years and described the importance of giving clear explanations of how it works to maximise older patients’ acceptance of this approach:

*‘I think, initially, they were quite reluctant. They* [older people] *were like, "*Well, no, I don't want to speak to them on the phone. I want to come in and see them.*" And so you have to explain that that’s, that’s possible, but initially they have to speak to them on the phone. And then, and then the doctor will organise that time for you to come in to see them on that day. So you had to, kind of, explain it a little bit more widely, in a, in a little bit more depth for them than you might have done a younger patient.’* (Receptionist, Practice 3)

For those patients whose practice was not already using telephone-first in March 2020, participants described, and seemed to accept, a change in expectations for receiving health care during the pandemic. They were experiencing a change in lifestyle, being at home more and so were better able to receive telephone calls. They experienced changes in attitudes towards social contact, preferring to avoid others:

*‘I’m absolutely fine about it, in fact it’s actually better 'cause it saves you the faff of going to the surgery and during these COVID times of course you’ve got the added jeopardy of catching COVID. So, if you don’t have to go to the surgery it takes that fear away.’* (Patient, F, 70−74 years, ≤3 conditions, White ethnicity, disability)

For a practice that was about to introduce telephone-first, COVID-19 provided a ‘cover’ for introducing the new service, but this meant patients did not necessarily expect it to continue after the pandemic:

‘*We were lucky in some ways that it was COVID as the reason. People understood why we were doing it*.’ (GP, Practice 7)

*‘I thought this was the, well, it was through the lockdown. I thought once we got the lockdown over, the coronavirus finished, it would go back to normal. I never dreamt that it would carry on with a telephone service*.’ (Patient, F, 75−79 years, ≤3 conditions, disability, White ethnicity)

While the pandemic acted as an impetus for service change in some practices, practice staff were having to evaluate the service as they went, while under pressure and with a constantly changing healthcare situation:

*‘I suppose in the, in the early stages we thought, “*Oh, COVID will end in a few months’ time, and we’ll be back to, we’ll be able to make a decision about what we do there and then*.”’* (GP, Practice 7)

The move to telephone-first meant there was sometimes the use of digital tools to supplement consultations, for example asking patients to send photographs by text. This extra dimension was challenging for some older patients:

*‘Eventually, it lasted, took about two weeks to get the photographs to her because of the breakdown on the, on the link with the, with the phone and stuff. So, that’s the problem with older people. I have an up-to-date, a mobile phone but it’s not one of these, you know, fandangle ones and fortunately, my brother’s got one but you know, it wasn’t, wasn’t convenient really.’* (Patient, M, 65−69 years, ≤3 conditions, White ethnicity)

For some staff, the key to having a positive telephone-first consultation was related to taking the time to explain why these additional elements were necessary:

*‘I think if you explain it* [talking about taking photographs to send to the practice]*, I suppose I think if people can do it with instruction, a lot of the people that have been more nervous, I’ve managed to win them over I suppose just what, what happened.’* (Advanced nurse practitioner, Practice 2)

Some practice staff discussed the importance of not making assumptions about older people and their ability to use the internet:

*‘I’ve just been pleasantly surprised about how they’ve all embraced it really. I think we, we probably haven’t given the older people credit where credit is due when it comes to digital things and, and changing their mindset and approaching things differently.’* (Practice nurse, Practice 5)

#### Conceptualising the consultation

Patients sometimes struggled to distinguish between triage and consultation, with negative views of the call-back crossing over into consultation:

*‘And I wonder if it would have been as long and drawn out if it hadn't been over the phone.’* (Patient, F, 65−69 years, ≤3 conditions, White ethnicity)

Telephone consultations were regarded by some patients or carers as inferior and associated with a lack of standard processes that normally typify the consultation, with face-to-face often regarded as superior. Being offered a face-to-face consultation was seen as validating a patient’s problem:

*‘But I’d still think the face-to-face is the best thing because you know I just do.’* (Patient, M, 65−69 years, ≤3 conditions, White ethnicity)

Practice staff were of the view that patients overall preferred or were accepting of the telephone-first system:

*‘ I think most people seem happy and if you reassure them you’ll see them or you will follow them up and there is a plan in place, they seem happy with it.’* (GP, Practice 5)

But in reality, the change to what constituted a consultation was difficult for some patients to adapt to.

#### Identities

##### Needs defined by age

There were a wide range of ages across the ‘older age’ participants, but there was still an identity associated with being older in itself.

Some participants felt they were viewed as less important or less deserving of treatment, or felt they were even treated differently, due to their age:

*‘One thing I do feel, generally, that when you're over 75, 80, you are perhaps not considered so important for treatment as if you were younger.’* (Patient, F, 70−74 years, ≤3 conditions, White ethnicity)

While some participants talked of feeling disregarded due to their age, general practice staff appeared aware of the difficulties these patients may have in accessing health care:

*‘There may still be patients out there who aren’t accessing our services, and therefore missing out on important care because they just don't know what, what’s going on. And if, if they’re older and not accessing the internet, I'm sure there’ll be some patients in that category.’* (GP, practice 7)

There was an awareness among practice staff that they may need to adapt their approach when communicating with older patients:

*‘I would say what it is, is about matching what the patient needs. So, either listening more, slowing down our speech, or, or shouting if they’re hard of hearing and that’s what they need on the phone.’* (Reception manager, Practice 7)

However, some participants had experienced their practice as not having made such adaptations:

*‘My one big gripe is that I don't hear very well and you can't use a hearing aid on the telephone. It doesn't work.’* (Patient, F, 70−74 years, ≤3 conditions, White ethnicity)

##### Identity as a carer

Some older people were responsible for caring for others, though did not necessarily regard themselves as formal carers. There was evidence of gender roles, with women being responsible for booking appointments in mixed-sex couples:

*‘So my wife picks up the phone and says, “I’ve booked you an appointment and the doctor’s ringing you back between two and six”, that’s it, it’s been done.’* (Patient, M, 75−79 years, ≥4 conditions, White ethnicity)

There were occasions when a patient needed to have a carer present for the telephone call, with practice staff in the focus groups recognising that family often played this important role.

*‘Families are very good and will often arrange a phone call for when they’re there.’* (Practice nurse, Practice 2)

### Trust as a driver for views and experiences

#### Trust in the system

Patients or carers who understood and trusted the telephone-first appointment system reported good experiences. They had trust that the system would provide the access they needed, when they needed it:

*‘It’s amazing how quickly you can get a response from a GP if you need one. I've never waited longer than perhaps a couple of hours.’* (Patient, M, ≥80 years, ≤3 conditions, mixed ethnicity)

#### Trust in practice staff

Patients or carers also needed to feel trust in the practice staff, trust that their problem would be addressed, that the health care they would receive would be appropriate to their needs. Continuity of care promoted trust but was not always available to patients and there was concern about what would happen to them if they did not know the doctor they were talking to:

*‘I’d prefer a doctor that I know understands my case who’s, who’s been with me on my journey, shall I say, and understands what my problems are, and knows whether to believe what I’m saying or not.’* (Patient, F, ≥80 years, ≥4 conditions, White ethnicity)

Conversely, some practice staff felt that telephone-first allowed them the opportunity to increase continuity of care to patients:

*‘I think one of the things we’ve done is we’ve really tried to improve continuity, we’ve had quite a drive at trying to improve continuity so that your clinical decision-making feels a little bit safer.’* (GP, Practice 1)

#### Trust in patients

Practice staff expressed a need to trust in patients using health care responsibly and to engage with the practice in a way that would allow them to receive the health care they needed, such as making themselves available for when they are called back:

*‘Practice nurses, we actually do book them a time… and we phone them at that time. So, if they’ve booked a pill check, we expect them to be on the end of the phone for the time they’re told.’* (Practice nurse, Practice 2)

### Impact on roles

#### Roles of practice staff

The concept of trust was very closely linked into the roles that patients or carers and practice staff play.

Patients felt that the role of the receptionist was to be warm and welcoming to patients trying to access their GP:

*‘And, and I think it’s important that their receptionists, or whoever it is, have compassion and understanding, you know, are not abrupt, or that they have a nice manner, basically… particularly with older people.’* (Patient, F, 70−74 years, ≤3 conditions, mixed ethnicity)

However, some patients or carers expressed concerns about reception staff and the requirement to provide them with personal medical information, not feeling that this was appropriate:

*‘Then you got the receptionist asking you what it’s for 'cause, I mean, sometimes it’s personal. It’s, sometimes, if it’s personal or it’s embarrassing you know, I don't like that.’* (Patient, M, 70−74 years, ≤3 conditions, White ethnicity)

Meanwhile reception staff described the importance of their role in establishing who needed a telephone call-back, allowing more efficiency, and reducing inappropriate telephone appointments with clinicians:

*‘We’re able to what I call bounce things off, so they don’t necessarily have to go to a clinician, we’ve got much more adept at doing that since March. And what that means is that whilst that’s additional workload for the reception team, and maybe the administration team, and the dispensary, that’s actually the way that it should be, so that there’s less inappropriate things going to a clinician.’* (Reception manager, Practice 7)

This suggested there were differing views and expectations about the systems between patients and general practices.

#### The patient role

Patients would describe themselves as the ‘good patient’;^22^ someone who does not ask for an appointment unless warranted and accepts the need to fit into the schedule of their GP. Patients demonstrated their respect for practice staff, that their health need was worthy, and that they were loyal to the general practice. Some patients seemed to put distance between themselves and other patients who they felt used health care unwisely:

*‘I mean, like I say I’ll tell you it’s very rare. I only ring the doctors if I class myself as really, really desperate….in a way, it’s like you need them to understand that if you are phoning it’s not, you’re not doing it lightly, you’re doing it because you’ve got a real reason to phone.’* (Patient, F, 65−69 years, ≥4 conditions, White ethnicity, disability, shielding during pandemic)

Some practice staff held generalised views about older patients, associated with behaviours such as not wanting to make a fuss about their health and using the time of healthcare professionals wisely:

*‘I would say I think the older people are better at answering their phones, probably ‘cause they’re maybe at home, you know, they’re … that traditional view of health care. Not, again, this probably isn’t the right thing to say, but I’m gonna say that respect around actually, you know, you, they’re a generation that remember parents paying for the doctor to come over and see them, where the younger generation don’t have that view.’* (Advanced nurse practitioner, Practice 2)

However, there were some practice staff who challenged assumptions about the age of patients and willingness to adapt to change:

*‘I’ve been pleasantly surprised how adaptable people have been. More so perhaps the tech bit in terms of maybe, not video particularly, now asking people to send in photographs and getting people to take the photograph, download it, send it to us, and then we have a further conversation. And very few have said they can’t do it, and we’re talking over six to eight months, so that’s been quite impressive, really. So, I guess maybe we’ve just underestimated people’s adaptability a little bit, you know*.’ (GP, Practice 5)

## Discussion

### Summary

Telephone-first did not look the same for all practices and their patients, and experiences of it varied. Older people and their practices had to adapt to the concept of the triage call-back, having to reconfigure what they understood to constitute a consultation. Trust between patients and their general practice influenced views and experiences, with acceptance of telephone-first linked to levels of trust in the general practice. There was sometimes a lack of communication about how the new system was intended to work and this influenced experiences negatively.

### Strengths and limitations

This study recruited a varied sample of practices allowing for variation within the sample. The matching of patients and practices allowed us to explore the views and experiences in both groups.

Recruiting practices that had implemented a telephone-first approach before March 2020 and others who had done so at the onset of COVID-19 pandemic allowed for the inclusion of a range of experience in delivery and implementation of the system. While the sample of included patients was varied and included patients from minority ethnic groups, we were unable to recruit patients of Black ethnic group (although no differences in views were observed based on ethnic group of participants). We also struggled to recruit participants that considered themselves to be carers, although the sample did include eight participants that made appointments for a family member.

The COVID-19 pandemic impacted on the study, as interviews and focus groups were conducted remotely due to restrictions. The Chief Investigator for the study was affected by COVID-19 related health concerns and these delayed the publication of this manuscript. However, we deemed the findings to be an important part of the record in how telephone-first works and, given the limited research specific to the telephone-first approach since 2019, we believe it to provide relevant evidence. Newer research has focused on remote consultation as a concept, amalgamating all types of telephone consultation and online consultation, and focusing on the remote nature.^13,23^ The telephone offers synchronous communication using fixed telephone lines as well as digital communication and, as such, deserves separate focus, especially in older people who are less likely to be using online forms of remote consultation and are more likely to use the telephone.^24^

### Comparison with existing literature

Previous research has found that older people do not consider telephone consultations a ‘medical act’.^25^ This resonates with our findings that conceptualising the consultation is difficult when using telephone consultation.^25^ We observed assumptions being made by practice staff about patients in older age groups in keeping with previous literature. A study examining how patients are supported to use online services in general practice found that practice staff routinely assumed that older people would not want to use online services and would find it difficult, but this was not borne out in reality, with factors including socioeconomic status having more influence on whether patients could use the services.^26^ Additionally, previous research examining remote consultations for asthma management found that older patients and patients with reduced mobility favoured the convenience and flexible access offered by remote consultations.^27^

Participants framed themselves as a ‘good patient’, a concept first described by Ziebland *et al,* ^22^ reflecting the ‘*ambivalence of health care seeking that individuals face in identifying when it is 'just right' to consult a general practitioner*’, with this ‘just right’ zone referred to as the ‘Goldilocks zone’. Ziebland *et al’*s work was not specific to older people and so it is not clear if our findings would apply to people in different age groups as well.

There has been limited research in recent years on telephone-first as an approach to delivering access, with a 2024 scoping review of access systems^28^ finding no new studies since 2019. Research published in 2022^29^ examined the impact of telephone triage (including but not limited to telephone-first) in people with multiple long-term conditions, but this study used data from 2011–2017 and a survey from Apr–Nov 2020, making it contemporaneous with our research. They found that there was no difference between those with or without multimorbidity in terms of time taken to see or speak to a GP when a telephone triage approach was in use. This concurred with our study, where views of patients were seemingly not influenced by their health status, which was varied in our sample.

### Implications for research and practice

This study provided insight into how older people experience telephone-first. It highlights the need for clear communication about how appointment systems work and how healthcare access needs will be met. This research provides evidence to guide the development of services that adequately fit the needs of older people. It is clear that systems implemented into practices need to be adequately explained, regarding the process, staff roles, and the expectations of patients, to allow for thorough understanding.

Patients in this study demonstrated their respect for practice staff, their ‘worthy’ health burden, and loyalty to the general practice. Some patients put distance between themselves and ‘bad’ patients, who they conceptualise as using health care unwisely.^30^ This attitude may be influencing how older people engage with access systems like telephone-first and should be taken into consideration.

Future research should explore the implications of telephone-first on the health outcomes of older patients, with a focus on the specific challenges faced by this population when it comes to access to general practice.

## Data

It is not possible to publish the data associated with this study, as this was not included in ethical applications or participant consent.
